# Associations between air pollution and daily outpatient visits for dry eye disease and the effect modification of temperature

**DOI:** 10.1186/s12889-025-22187-4

**Published:** 2025-03-27

**Authors:** Dandan Chu, Juan Chen, Chenlu Yang, Yan Li, Mingwei Wang, Junzhe Bao

**Affiliations:** 1 College of Public Health, Zhengzhou University, Zhengzhou, 450001 China; 2 Henan Eye Hospital, Henan Provincial People’s Hospital, People’s Hospital of Zhengzhou University, Zhengzhou, 450003 China; 3 Department of Ophthalmology, the Affiliated Hospital of Hangzhou Normal University, Hangzhou, 310015 China; 4 Department of Infection Control, Henan Provincial People’s Hospital, People’s Hospital of Zhengzhou University, Zhengzhou, 450003 China; 5 Department of Cardiology, the Affiliated Hospital of Hangzhou Normal University, Zhejiang Key Laboratory of Medical Epigenetics, Hangzhou Normal University, Hangzhou, 310015 China

**Keywords:** Air pollution, PM_2.5_ constituents, Modification effect, Dry eye disease

## Abstract

**Background:**

Dry eye disease (DED) is one of the most common ocular surface disorders caused by various contributors. Air pollutants are considered a risk factor for ocular surface diseases. We aimed to investigate the associations between air pollutants (PM_2.5_, PM_10_, NO_2_, SO_2_, CO and O_3_) and PM_2.5_ constituents and daily outpatient visits for DED, as well as the modifying effect of temperature on the associations.

**Methods:**

Daily data on DED outpatient visits and environmental variables during 2014–2019 were collected in Hangzhou, China. Distributed lag nonlinear models (DLNM) combined with time-stratified case-crossover design were utilized to evaluate the effects of air pollutants and PM_2.5_ constituents on DED daily outpatient visits during 0‒3 lag days. Furthermore, we also estimated the modification effect of temperature stratified by median. The attributable fraction (AF) of air pollutants and PM_2.5_ constituents on DED outpatient visits were quantified. Stratified analyses of gender, age, and seasons were conducted to assess vulnerable population characteristics and high-risk periods.

**Results:**

Every interquartile range increase in PM_2.5_, PM_10_, NO_2_, SO_2_ and CO concentration were significantly associated with daily DED cases. The AF were 6.42% (95% CI: 1.09%, 11.58%), 8.00% (2.60%, 13.60%), 18.65% (11.52%, 25.21%), 10.82% (3.92%, 17.24%) and 12.28% (0.23%, 22.86%), respectively. For PM_2.5_ constituents, NO_3_^−^ and NH_4_^+^ were associated with DED, with AF of 4.34% (0.21%, 8.11%) and 4.84% (0.18%, 9.09%), respectively. The effects of air pollution were significant in low-temperature level for PM_2.5_, PM_10_, NO_2_, SO_2_, and CO; while the effects were statistically insignificant in high-temperature level. Subgroup analyses indicated significant associations were present in winter and among patients aged 21–40 but insignificant in other seasons and age groups.

**Conclusion:**

Our results revealed that air pollutants were associated with DED outpatient visits. Low temperatures might increase the hazardous effects of air pollution. Besides, individuals aged 21–40 were vulnerable to air pollution, and winter was the high-risk period.

**Clinical trial number:**

Not applicable.

**Graphical abstract:**

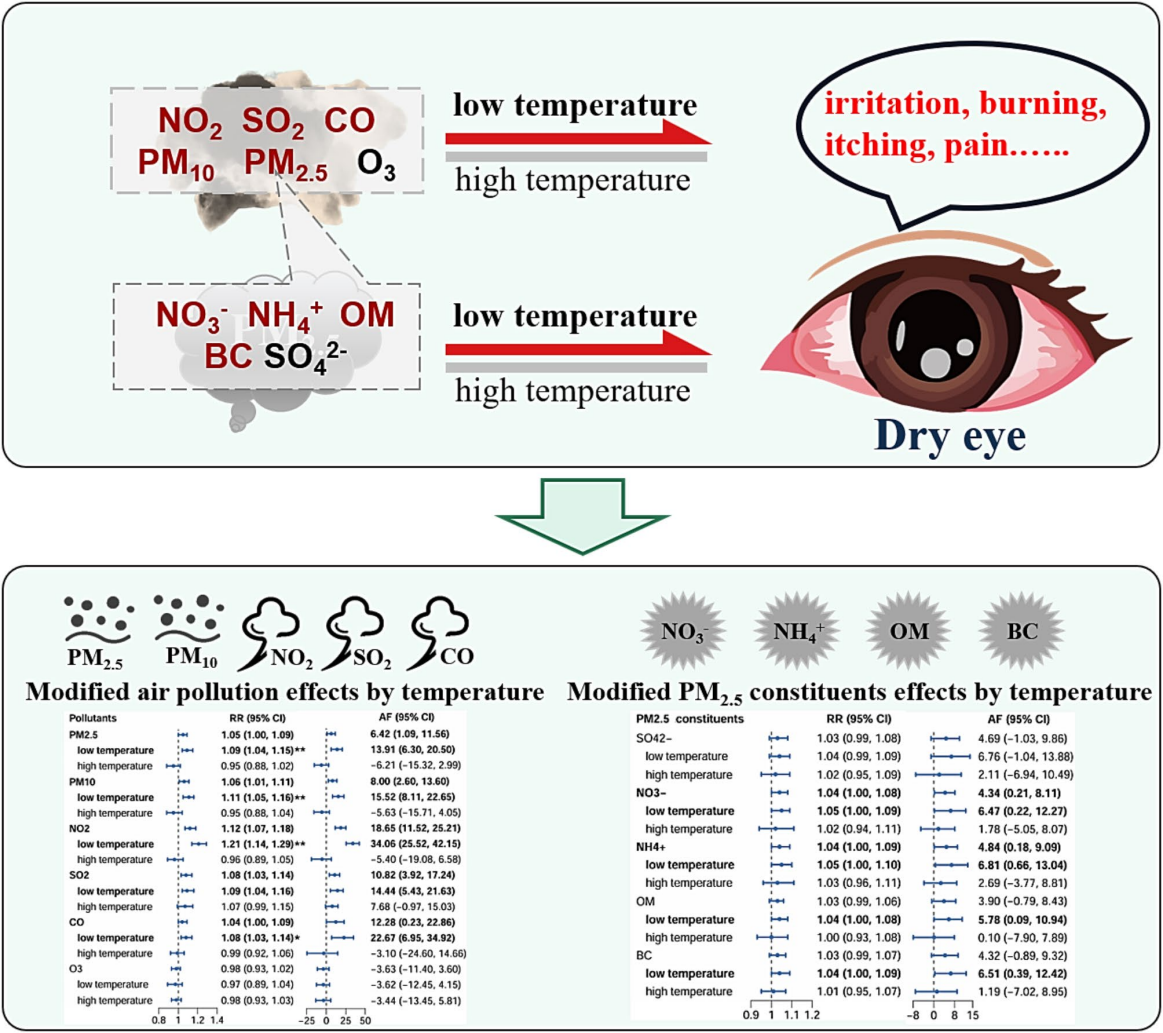

**Supplementary Information:**

The online version contains supplementary material available at 10.1186/s12889-025-22187-4.

## Introduction

Dry eye disease (DED) is a chronic and multifactorial ophthalmic disorder characterized by tear film instability, vicious cycle of inflammation and ocular microenvironment imbalance. It may be accompanied by visual disturbances and ocular discomforts, such as dryness, itching, irritation, burning, and pain, adversely affecting patients’ visual acuity and life quality [1–3]. The rising incidence of DED represents a major global health issue, posing massive economic and medical burdens to society [4]. It is reported that one to two out of every five people suffer from dry eye, with a morbidity of about 21–30% in China [5]. The pathogenesis and progression of DED are intricately linked to immunity, metabolism, hormones, and environmental factors, including humidity, temperature, and air pollution [6, 7]. Regular exposure to air contaminants has been identified as a contributing factor that enhances the susceptibility of the ocular surface to these harmful substances [8].

Despite multiple investigations into the association between air pollution and DED outpatient visits, there was still a lack of consistency in methodology and conclusions. A study conducted in Shenzhen using time series analysis found that PM_2.5_, PM_10,_ NO_2_, and O_3_ could facilitate the development of DED for children, as inferred from generalized additive models [9]. A positive correlation between PM_10_, O_3_, CO and temperature with the incidence of DED in Changchun, China, was identified using correlation, binary nonconditional logistic regression and machine learning methods [5]. A cross-sectional study conducted in South Korea revealed that reduced humidity levels and elevated ozone concentrations were associated with an increased risk of DED using multivariate logistic regression analysis [10]. Cross-sectional designs and conventional regression models (e.g., logistic regression) are commonly used to explore air pollution’s medium- and long-term health effects (e.g., associations at the monthly or yearly level) [5, 10]. In contrast, time-series or time-stratified case-crossover designs are commonly used to explore the short-term health effects of air pollution (e.g., associations at the daily level) [11, 12].

Previous studies revealed that PM_2.5_ was associated with DED outpatient visits [9, 13–15]. The likely reason was that elevated PM_2.5_ concentration increased tear osmolarity, inflammatory cytokines expression, and inflammatory cell infiltration, induced corneal epithelial cell apoptosis, resulting in DED [5, 16, 17]. The total mass of PM_2.5_, roughly used as an indicator for total PM_2.5_ exposure, might underestimate the combined effects of the various constituents of PM_2.5_ on pediatric health outcomes, including anemia, acute respiratory infections, and low birth weight [18]. Therefore, the actual aggregate effect of PM_2.5_ on health is anticipated to exceed the assessments based on total PM_2.5_ mass alone. PM_2.5_ is a complex mixture containing numerous chemical constituents with diverse origins and toxicity levels. These components, originating from varied sources, could generate distinct health effects. For instance, substances such as ammonium, nitrate, and bromine, which are commonly linked to traffic emissions, have been positively related to an elevated risk of preterm birth [19]. Black carbon particles from biomass and fossil fuel burning are associated with acute ischemic stroke [20]. Identifying the association of PM_2.5_ constituents with the incidence of DED could identify the most hazardous PM_2.5_ constituents and take actions to control their contributing sources and reduce vulnerable populations’ exposure to more toxic species. At present, the association of PM_2.5_ constituents with DED outpatient visits is unclear.

Several studies have found that temperature and air pollution were independently associated with DED morbidity [5, 9, 15]. Additionally, temperature was found to modify the association of air pollution with cardiovascular disease and periodontal disease [21, 22]. However, the modifying effect of temperature on the association between air pollution and dry eye is currently unclear.

This study explored the association between exposure to air pollutants and PM_2.5_ constituents with DED daily outpatient visits, analyzed the modification effect of air temperature on the association, and identified the associated vulnerable populations and high-risk periods.

## Methods

### Outpatient data

Medical records of outpatient visits covering the period from July 1, 2014 to December 31, 2019 were retrieved from the Eye Centre of the First Affiliated Hospital of Hangzhou Normal University (Hangzhou, Zhejiang Province, China). The scope of the investigation was limited to individuals who were first-time patients within the permanent resident population of Hangzhou and who had been diagnosed with DED. Patient data collected included demographic details such as patient ID, visit date, specific diagnosis, age, gender and home address.

### Air pollution and meteorology data

Daily meteorological data were obtained from the Hangzhou station of the National Meteorological Data Sharing Platform (http://data.cma.cn/). This source provided comprehensive information, including mean temperature, relative humidity, atmospheric pressure, rainfall, wind speed, and duration of sunshine. For air quality assessment, daily average concentrations of PM_2.5_, PM_10_, NO_2_, SO_2_, CO, and 8-h maximum levels of O_3_ were obtained from 11 fixed air pollution monitoring stations distributed throughout Hangzhou. PM_2.5_ component data (black carbon [BC], organic matter [OM]), nitrate [NO_3_^−^], sulfate [SO_4_^2−^], and ammonium [NH_4_^+^]) were obtained from the Tracking Air Pollution in China (TAP) dataset (http://tapdata.org.cn/).

### Statistical analysis

A descriptive analysis was conducted for DED outpatient visits, air pollutants, PM_2.5_ constituents, temperature, and relative humidity. The distribution of outpatients was analyzed across different genders, age groups, and seasons. The distributions of air pollutants, PM_2.5_ constituents, temperature, and relative humidity were examined (mean, SD, min, median, max, et al.). Spearman’s rank correlation coefficient was utilized to assess the correlation among air pollutants, meteorological factors and daily DED cases.

A time-stratified case-crossover design with a distributed lag nonlinear model (DLNM) based on a conditional quasi-Poisson generalized linear regression model was employed to assess the association between air pollutants and daily outpatient visits for DED. Time-stratified case-crossover designs are akin to case-control studies matched by time stratum, define the onset date of DED outpatient visits as the case period and the remaining days within the same year, month, and day of the week as the control periods, with each case period matched to multiple control periods. This design has been widely used in environmental epidemiological studies and can control individual characteristics, long-term trends, seasonal variations, and day-of-the-week effects [5, 23]. We evaluated the effects of air pollutants on DED daily outpatient visits through DLNM [24]. In this study, the maximum lag days of three days were chosen based on the distribution of different lag time effects and previous research [16]. The natural cubic spline function was applied to control the relative humidity and temperature with 3 degrees of freedom (df).

We next investigated the modifying effect of temperature on the associations. An interaction term, constructed from a cross-basis matrix related to air pollution and a temperature stratification indicator, was included in the model to assess the effect of air pollutants on DED daily outpatient visits at different temperature levels. Temperature was categorized as low or high based on the median value.

Relative risk (RR) with 95% confidence intervals was employed to evaluate the cumulative risk of outpatient visits for DED associated with each interquartile range (IQR) increment in air pollutants concentration and their possible interactions with different temperature levels. The attributable fraction of DED outpatient visits attributable to air pollutants was calculated using previously described methods [25]. In brief, the cumulative relative risk corresponding to each day’s air pollutants was used to calculate the AF. The 95% empirical confidence interval (eCI) for AF were calculated using Monte Carlo simulations [24]. Furthermore, the Cochran Q test was utilized to compare statistical differences between the low and high-temperature level.

### Subgroup analysis

Subgroup analysis were performed to evaluate vulnerable population characteristics and high-risk periods based on gender (male and female), age groups (0–20, 21–40, 41–60, and ≥ 61 years), and season (March-May in spring, June-August in summer, September-November in autumn, and December, January, and February in winter). The subgroup analysis used the same statistical models as the main analysis.

### Sensitivity analysis

The robustness was confirmed through sensitivity analyses, which were carried out by altering the maximum lag days and changing the df values for temperature and relative humidity. The statistical analyses were conducted utilizing R software (version 4.3.1), employing the package “dlnm” to calculate the effects of air pollutants on DED outpatient visits.

## Results

### Data description

The descriptive statistics information was presented in Table 1. Altogether, 29,933 DED patients were included. The majority of dry eye patients were female, accounting for 63.1% (*n* = 18,901) of enrolled patients. In addition, patients aged 21–40 (*n* = 9,928; 33.2%) and 41–60 (*n* = 10,395; 34.7%) years, which together represented 67.9% (*n* = 20,323) of the total patient population. The average number of DED cases per day was approximately 14. DED outpatient visits were highest in spring and lowest in winter. During the study period, the average concentrations of PM_2.5_, PM_10_, NO_2_ in Hangzhou were 46.35 µg/m^3^, 74.87 µg/m^3^, 41.82 µg/m^3^, respectively, which were higher than the Level 2 of the ambient air quality standards in China [35 µg/m^3^ (PM_2.5_), 70 µg/m^3^ (PM_10_), and 40 µg/m^3^(NO_2_)]. In addition, the daily mean temperature and relative humidity were recorded as 17.95 °C and 73.75%, respectively. The Spearman correlation analysis among air pollutants, meteorological variables, and daily DED cases was presented in Table S1.

**Table 1:** Distribution of daily DED outpatient visits, air pollutants and meteorological factors

Variables	Mean	SD	Min	*P*_25_	*P*_50_	*P*_75_	Max
**DED cases (n = 29, 933)**							
**Gender (n)**							
Male (*n* = 11,032)	5	3	0	3	5	7	19
Female (*n* = 18,901)	9	5	0	5	8	11	28
**Age (years**,** n)**							
0–20 (*n* = 1,762)	1	1	0	0	0	1	8
21–40 (*n* = 9,928)	5	3	0	2	4	6	18
41–60 (*n* = 10,395)	5	3	0	3	4	6	24
61- (*n* = 7,848)	4	3	0	2	3	5	16
**Season (n)**							
Spring (*n* = 8,016)	15	7	0	9	14	19	41
Summer (*n* = 7,744)	14	7	2	9	13	18	39
Autumn (*n* = 7,441)	14	7	0	9	12	18	39
Winter (*n* = 6,732)	12	8	0	7	11	17	44
**Air pollutants**							
PM_2.5_ (µg/m^3^)	46.35	28.08	5	26.68	40	59	224
PM_10_ (µg/m^3^)	74.87	40.28	8.73	45	67	96	283
NO_2_ (µg/m^3^)	41.82	16.10	6.82	30	40	52	110
SO_2_ (µg/m^3^)	11.62	7.23	3	7	9	14	78
CO (mg/m^3^)	0.85	0.23	0.41	0.69	0.81	0.97	2.04
O_3_ (µg/m^3^)	62.73	37.34	5	36	56	82	233.45
**PM**_**2.5**_ **constituents**							
SO_4_^2−^ (µg/m^3^)	6.68	3.40	0.45	4.15	6.10	8.49	26.32
NO_3_^−^ (µg/m^3^)	7.56	5.50	0.50	3.58	6.24	10.01	41.53
NH_4_^+^ (µg/m^3^)	5.31	3.46	0.32	2.74	4.63	7.04	26
OM (µg/m^3^)	7.86	4.84	0.43	4.54	6.81	9.84	43.71
BC (µg/m^3^)	1.53	0.84	0.07	0.95	1.34	1.94	6.30
**Meteorological factors**							
Temperature (°C)	17.95	8.66	-5	10.3	19	24.9	35.6
Relative humidity (%)	73.75	13.92	27	64	75	85	98

PM_2.5_, fine particulate matter; PM_10_, inhalable particulate matter; O_3_, ozone; NO_2_, nitrogen dioxide; SO_2_, sulfur dioxide; CO, carbon monoxide

### Associations between air pollutants and DED daily outpatient visits and the effect modification of temperature

**Fig. 1:**
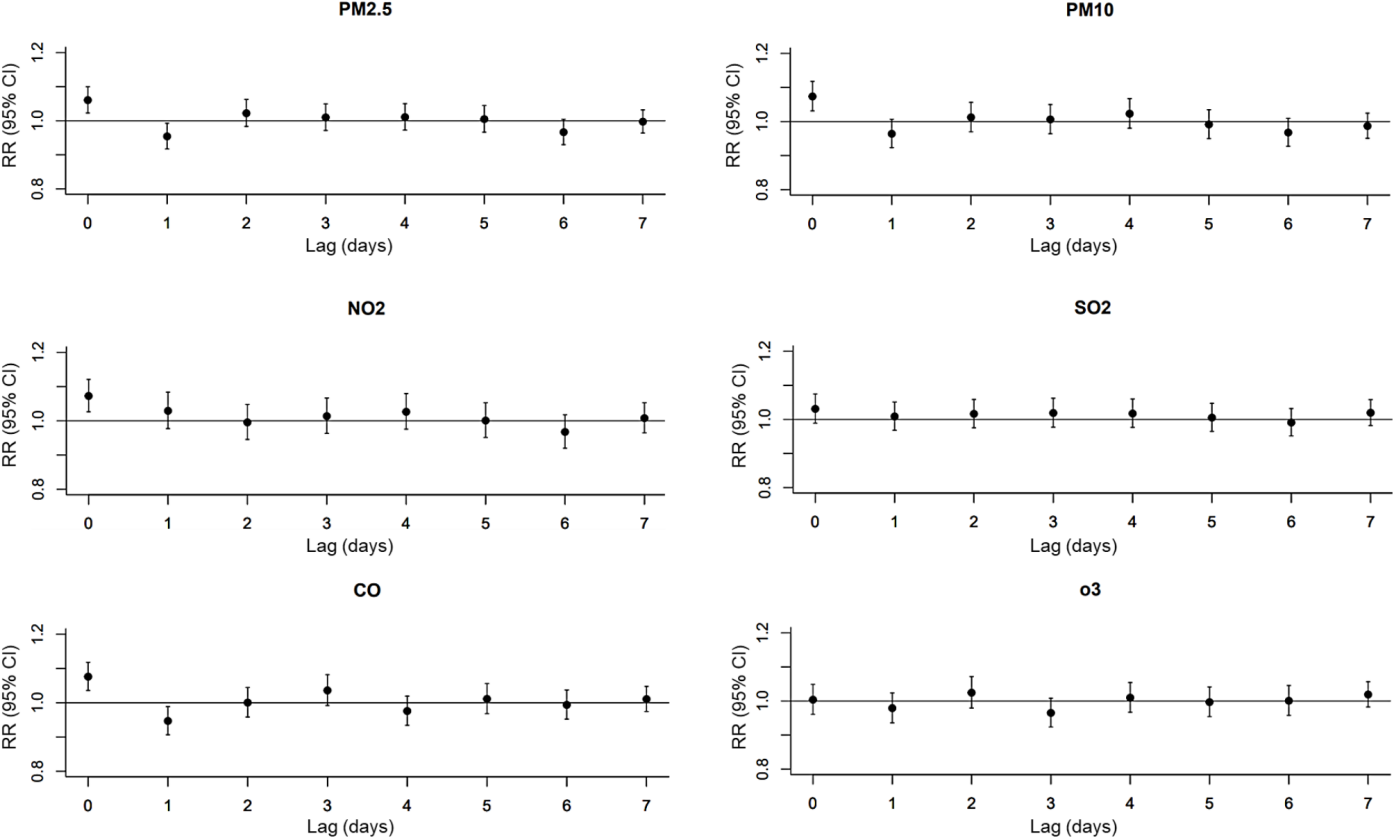
RR (95% CI) of air pollution on DED outpatient visits at different lag days (0–7)

**Fig. 2:**
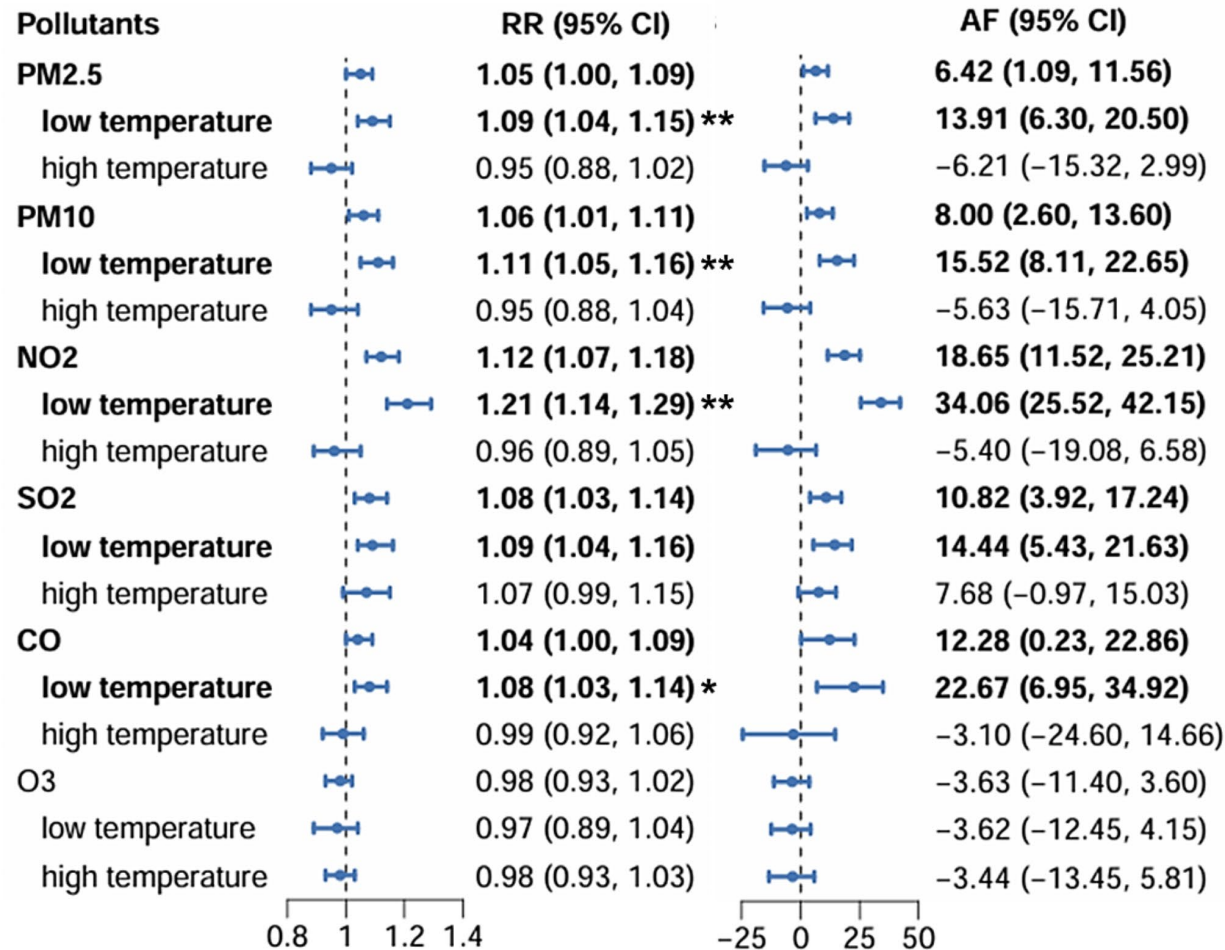
The RR and AF (95% CI) for DED outpatient visits per IQR increase in air pollution concentration and the effect modification across different temperature levels. The differences of RR between low and high temperature were tested by Cochran Q test. ^*^P < 0.05, ^**^P < 0.01

The effects of single-day lagged air pollutants exposure were estimated for 0-, 1-, 2-, 3-, 4-, 5-, 6-, and 7-day lags (Fig. 1). The hazardous effects of air pollutants mainly occurred within a 3-day period. Therefore, Lags of 0–3 days were chosen to assess potential cumulative exposure risk. Significant associations were identified between every interquartile range increment in air pollutants (PM_2.5_, PM_10_, NO_2_, SO_2_ and CO) concentration and daily DED cases during the 0–3 day lag period, with relative risk (RR) of 1.05 (1.00, 1.09), 1.06 (1.01, 1.11), 1.12 (1.07, 1.18), 1.08 (1.03, 1.14), and 1.04 (1.00, 1.09), respectively. The AF of PM_2.5_, PM_10_, NO_2_, SO_2_ and CO were 6.42% (1.09%, 11.58%), 8.00% (2.60%, 13.60%), 18.65% (11.52%, 25.21%), 10.82% (3.92%,17.24%) and 12.28% (0.23%, 22.86%), respectively (Fig. 2).

The cumulative risk of air pollutants on DED might be modified by temperature levels (Fig. 2). Notably, exposure to PM_2.5_, PM_10_, NO_2_, SO_2_, and CO at low temperatures were associated with DED incidence. The RR of PM_2.5_, PM_10_, NO_2_, SO_2_, and CO at low temperatures induced DED were 1.09 (1.04, 1.15), 1.11 (1.05,1.16), 1.21 (1.14, 1.29),1.09 (1.04,1.16) and 1.08 (1.03,1.14), respectively. The AF were 13.91% (6.30%, 20.50%), 15.52% (8.11%, 22.65%), 34.06% (25.52%, 42.15%), 14.44% (5.43%, 21.63%), and 22.67% (6.95%, 34.92%), respectively (Fig. 2).

### Associations between PM_2.5_ constituents and DED daily outpatient visits and the effect modification of temperature

**Fig. 3:**
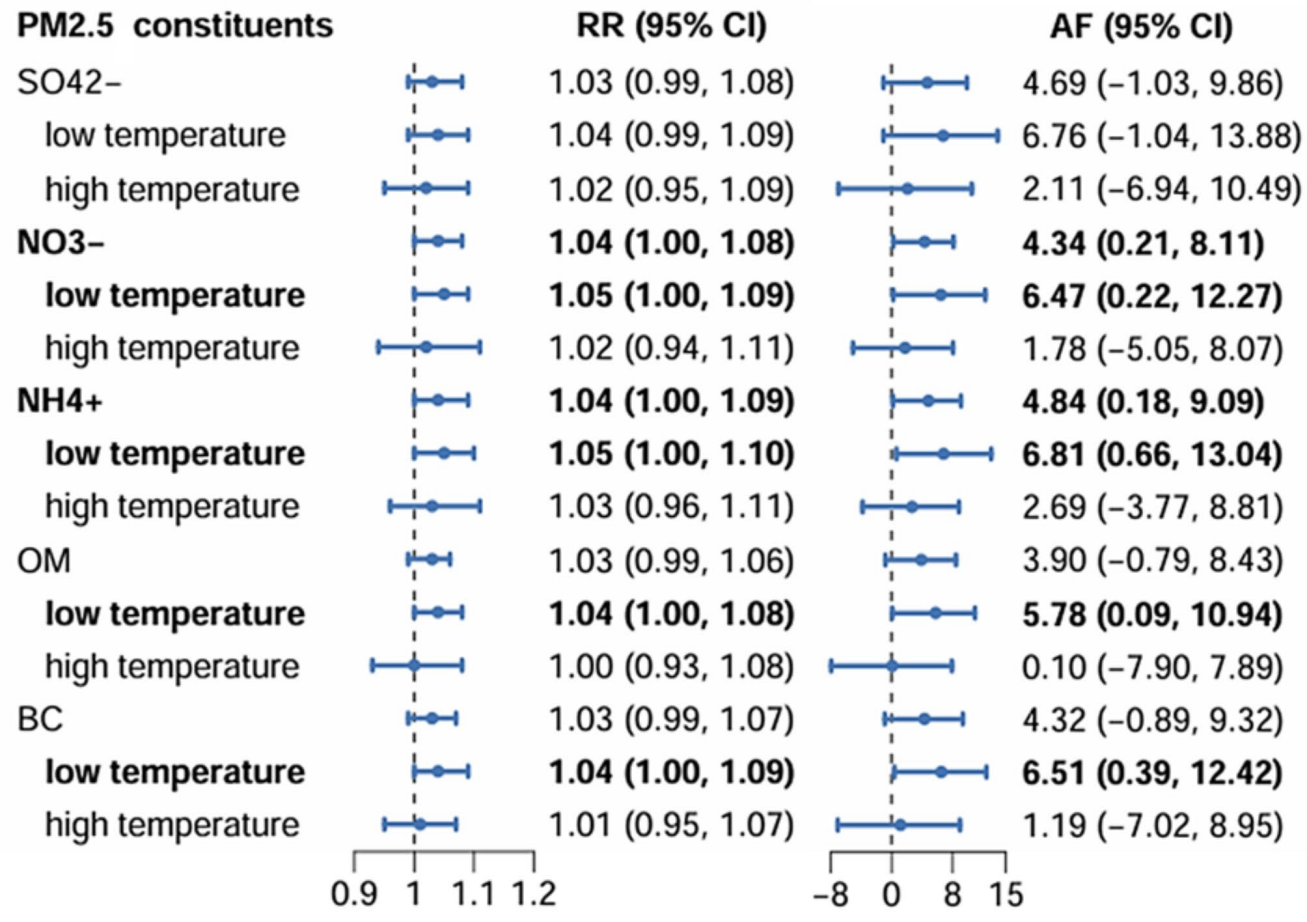
The RR and AF (95% CI) for DED outpatient visits per IQR increase in PM_2.5_ constituent concentration and the effect modification across different temperature levels

We further explored the associations of PM_2.5_ constituents on DED daily outpatient visits (Fig. 3). NO_3_^−^ and NH_4_^+^ were associated with DED. The RR were 1.04 (1.00, 1.08) for NO_3_^−^ and 1.04 (1.00, 1.09) for NH_4_^+^, respectively. The AF were 4.34% (0.21%, 8.11%) for NO_3_^−^ and 4.84% (0.18%, 9.09%) for NH_4_^+^, respectively. Low temperature enhanced the effects of NO_3_^−^, NH_4_^+^, OM and BC on DED outpatient visits. The RR were 1.05 (1.00,1.09) for NO_3_^−^, 1.05 (1.00,1.10) for NH_4_^+^, 1.04 (1.00, 1.08) for OM and 1.04 (1.00, 1.09) for BC at low-temperature conditions, respectively. The AF were 6.47% (0.22%, 12.27%) for NO_3_^−^, 6.81% (0.66%, 13.04%) for NH_4_^+^, 5.78% (0.09%, 10.94%) for OM and 6.51% (0.39%, 12.42%) for BC at low-temperature level, respectively (Fig. 3).

### Subgroup analysis

Examination of vulnerable demographic subgroups revealed that PM_2.5_, PM_10_ and CO had significant effects on males and people aged 21–40. For NO_2_, significant associations were observed in both male and female populations as well as people older than 20. The effect of SO_2_ was found to be significant in both male and female populations as well as people aged 21–40. The effects of these six air pollutants were all significant in winter. However, O_3_ is a protective factor in winter and people younger than 21. PM_2.5_, PM_10_, NO_2_, SO_2_ and CO were all significant for both males and people aged 21–40 at low temperatures (Table 2).

**Table 2:** Subgroup analyses stratified by patient characteristics

	PM_2.5_	PM_10_	NO_2_	SO_2_	CO	O_3_
**Gender**						
**Male**	**1.07 (1.01**,** 1.13)**	**1.07 (1.01**,** 1.13)**	**1.14 (1.08**,** 1.22)**	**1.09 (1.02**,** 1.16)**	**1.06 (1.00**,** 1.12)**	0.99 (0.93, 1.05)
low temperature	**1.10 (1.03**,** 1.16)**	**1.10 (1.04**,** 1.17)**	**1.23 (1.14**,** 1.33)**	**1.13 (1.05**,** 1.22)**	**1.10 (1.02**,** 1.18)**	0.98 (0.88, 1.08)
high temperature	0.99 (0.89, 1.10)	0.97 (0.87, 1.08)	0.99 (0.89, 1.10)	1.01 (0.91, 1.11)	1.01 (0.92, 1.10)	1.00 (0.94, 1.07)
**Female**	1.02 (0.97, 1.07)	1.02 (0.97, 1.07)	**1.10 (1.04**,** 1.16)**	**1.08 (1.02**,** 1.14)**	1.04 (0.99, 1.09)	0.97 (0.92, 1.02)
low temperature	1.05 (0.99, 1.10)	1.05 (0.99, 1.10)	**1.19 (1.11**,** 1.27)**	**1.07 (1.01**,** 1.14)**	**1.08 (1.01**,** 1.15)**	0.96 (0.88, 1.05)
high temperature	0.94 (0.86, 1.03)	0.93 (0.85, 1.02)	0.95 (0.87, 1.04)	**1.10 (1.01**,** 1.20)**	0.98 (0.91, 1.06)	0.97 (0.92, 1.03)
**Age**						
**< 21**	1.00 (0.88, 1.15)	0.97 (0.85, 1.10)	1.08 (0.92, 1.25)	1.15 (0.97, 1.36)	1.12 (0.97, 1.29)	**0.81 (0.70**,** 0.94)**
low temperature	1.02 (0.88, 1.18)	1.01 (0.88, 1.16)	1.16 (0.97, 1.39)	1.06 (0.88, 1.28)	1.17 (0.99, 1.40)	0.86 (0.69, 1.08)
high temperature	0.89 (0.69, 1.15)	0.80 (0.62, 1.03)	0.93 (0.73, 1.19)	1.23 (0.99, 1.53)	1.02 (0.83, 1.26)	**0.78 (0.67**,** 0.90)**
**21–40**	**1.07 (1.01**,** 1.14)**	**1.07 (1.01**,** 1.14)**	**1.18 (1.10**,** 1.26)**	**1.12 (1.04**,** 1.21)**	**1.07 (1.00**,** 1.14)**	0.96 (0.89, 1.03)
low temperature	**1.11 (1.04**,** 1.19)**	**1.12 (1.05**,** 1.19)**	**1.32 (1.22**,** 1.44)**	**1.16 (1.07**,** 1.26)**	**1.16 (1.07**,** 1.25)**	**0.88 (0.78**,** 0.99)**
high temperature	0.95 (0.84, 1.07)	0.92 (0.82, 1.04)	0.92 (0.81, 1.04)	1.06 (0.95, 1.18)	0.93 (0.84, 1.03)	0.98 (0.91, 1.06)
**41–60**	1.02 (0.96, 1.08)	1.02 (0.96, 1.08)	**1.09 (1.02**,** 1.16)**	1.05 (0.98, 1.13)	1.03 (0.97, 1.09)	1.01 (0.94, 1.07)
low temperature	1.04 (0.97, 1.10)	1.04 (0.97, 1.11)	**1.16(1.08**,** 1.26)**	**1.07 (1.00**,** 1.16)**	1.03 (0.96, 1.11)	1.02 (0.92, 1.14)
high temperature	0.96 (0.86, 1.07)	0.97 (0.87, 1.08)	0.96 (0.86, 1.07)	1.02 (0.92, 1.13)	1.02 (0.93, 1.13)	1.00 (0.94, 1.08)
**> 60**	1.03 (0.96, 1.10)	1.03 (0.96, 1.10)	1.08 (1.00, 1.16)	1.05 (0.97, 1.14)	1.02 (0.95, 1.09)	1.01 (0.94, 1.08)
low temperature	1.05 (0.97, 1.13)	1.05 (0.97, 1.13)	**1.11 (1.01**,** 1.22)**	1.04 (0.94, 1.13)	1.03 (0.94, 1.13)	1.04 (0.92, 1.18)
high temperature	0.99 (0.88, 1.11)	0.98 (0.87, 1.11)	1.03 (0.91, 1.17)	1.10 (0.98, 1.24)	1.01 (0.91, 1.12)	1.01 (0.93, 1.09)
**Season**						
**Spring**	0.92 (0.81, 1.03)	0.96 (0.87, 1.05)	0.96 (0.88, 1.04)	1.04 (0.93, 1.16)	0.95 (0.87, 1.04)	1.00 (0.91, 1.10)
low temperature	0.92 (0.79, 1.08)	1.01 (0.89, 1.16)	1.00 (0.90, 1.12)	1.05 (0.92, 1.20)	1.00 (0.88, 1.13)	1.08 (0.95, 1.24)
high temperature	0.92 (0.79, 1.07)	0.91 (0.80, 1.03)	0.91 (0.81, 1.02)	1.04 (0.92, 1.17)	0.90 (0.80, 1.02)	0.96 (0.86, 1.06)
**Summer**	0.94 (0.87, 1.02)	1.02 (0.94, 1.10)	1.00 (0.93, 1.08)	1.00 (0.93, 1.07)	1.00 (0.94, 1.07)	1.05 (0.99, 1.12)
low temperature	0.96 (0.87, 1.06)	1.00 (0.91, 1.10)	0.97 (0.89, 1.06)	1.00 (0.93, 1.08)	1.00 (0.92, 1.09)	1.04 (0.94, 1.14)
high temperature	0.93 (0.83, 1.05)	1.04 (0.94, 1.15)	1.03 (0.93, 1.14)	0.99 (0.91, 1.08)	1.00 (0.92, 1.10)	1.07 (0.99, 1.15)
**Autumn**	1.02 (0.91, 1.13)	1.05 (0.96, 1.15)	1.05 (0.96, 1.14)	1.03 (0.94, 1.13)	1.07 (0.98, 1.16)	0.95 (0.87, 1.04)
low temperature	1.09 (0.95, 1.25)	1.10 (0.99, 1.22)	1.12 (0.99, 1.26)	0.99 (0.89, 1.10)	1.08 (0.96, 1.20)	0.99 (0.88, 1.12)
high temperature	0.94 (0.82, 1.09)	0.94 (0.82, 1.09)	1.02 (0.91, 1.15)	1.07 (0.97, 1.19)	1.03 (0.93, 1.14)	0.96 (0.88, 1.06)
**Winter**	**1.13 (1.03**,** 1.24)**	**1.15 (1.05**,** 1.26)**	**1.38 (1.26**,** 1.52)**	**1.28 (1.14**,** 1.45)**	**1.17 (1.07**,** 1.29)**	**0.76 (0.69**,** 0.85)**
low temperature	**1.18 (1.03**,** 1.35)**	**1.16 (1.01**,** 1.34)**	**1.40 (1.21**,** 1.62)**	**1.20 (1.05**,** 1.38)**	**1.25 (1.09**,** 1.43)**	**0.76 (0.66**,** 0.87)**
high temperature	1.02 (0.90, 1.16)	1.05 (0.93, 1.20)	**1.35 (1.20**,** 1.51)**	**1.26 (1.10**,** 1.45)**	1.05 (0.93, 1.19)	**0.77 (0.69**,** 0.86)**

### Sensitivity analysis

Sensitivity analysis demonstrated that the low-temperature enhanced PM_2.5_, PM_10_, NO_2_, SO_2_ and CO effects on DED daily outpatient visits were robust after adjusting the cumulative lag days from 2 to 5 and changing the df values for temperature and relative humidity from 2 to 4 (Table S2, S3 and S4).

## Discussion

To our knowledge, this research is pioneering in demonstrating the modifying effect of temperature on the associations between air pollution (including PM_2.5_ constituents) and DED outpatient visits. In addition, we have also provided insights into the associated attributable risks, the characteristics of the vulnerable population and the peak risk periods. Significant associations were observed between PM_2.5_, PM_10_, NO_2_, SO_2_ and CO and DED outpatient visits. Besides, NO_3_^−^ and NH_4_^+^ of PM_2.5_ constituents were associated with DED. We found that low temperatures might enhance the effects of PM_2.5_, PM_10_, NO_2_, SO_2_ and CO, and individuals aged 21–40 were potentially vulnerable populations. The seasonal pattern of air pollutant-related DED outpatient visits revealed a stronger association during the winter compared to other seasons.

In this study, the effects of multiple air pollutants were examined. We found that PM_2.5_, PM_10_, NO_2_, SO_2_ and CO were significantly associated with daily DED cases, findings that were consistent with those reported in prior research [5, 9, 10]. The risk of DED morbidity rose with higher levels of air pollutants. Notably, our study identified more types of air pollutants associated with DED. This could be attributed to the time-stratified case-crossover design utilized in our research, which controlled for long-term trends, seasonal variations and potential confounders such as patients’ underlying socioeconomic conditions, education level, living arrangements, and morbid states during the analysis, resulting in different outcomes for each ambient air pollutant on the DED morbidity [5, 10]. Furthermore, our study was conducted on all age groups, not on children [9]. In conclusion, our findings further supported that air pollution exposure increased the risk of DED outpatient visits. The underlying mechanism by which air pollution contributes to the development of dry eye is thought to involve a complex cascade of events. Specifically, PM_2.5_ and PM_10_ have been reported to alter the precorneal tear film (PTF), and NO_2_, SO_2_, and CO can alter the structural composition of the outermost lipid layer of the PTF, thereby causing ocular irritation and inflammation [15]. Besides, exposure to air pollutants is thought to trigger an overproduction of reactive oxygen species (ROS) in ocular surface epithelial cells [26]. Excessive ROS disrupt antioxidant defenses, causing oxidative damage and epithelial cell dysfunction, which initiate inflammatory pathways and contribute to dry eye [27]. Therefore, understanding the precise mechanisms by which air pollution induces dry eye is crucial for developing effective preventive and therapeutic strategies to reduce the risk and severity of dry eye in polluted environments.

No significant association was observed between O_3_ exposure and DED outpatient visits in the present study, consistent with previous reports from Hangzhou and Taiwan [15, 28]. While several studies have suggested that O_3_ was associated with DED due to its primary irritant properties. It has been proposed that subclinical ocular inflammation resulting from ozone exposure may contribute to the development of DED [29–31]. However, it is important to note that O_3_ also has beneficial effects. O_3_ possesses strong antioxidant capacity and anti-inflammatory activity, which can attenuate ischemic damage in the retina, promote tissue repair and offer neuroprotection [32–36]. Additionally, the concentration of O_3_ in our study was lower than that reported in Shenzhen [9]. Our seasonal analysis revealed a protective effect of O_3_ in winter, possibly due to the dependence of O_3_ formation on nitrogen oxides, hydrocarbons, and sunlight, leading to higher levels in warm seasons. Thus, the observed protective effect of O_3_ on DED was more significant in winter compared to other seasons.

PM_2.5_, a composite mixture containing diverse constituents with varying sources and toxicities, can worsen ocular discomfort and inflammation, ultimately leading to the diagnosis of DED [37]. This study showed that NO_3_^−^ and NH_4_^+^ were statistically associated with the risk of DED. Significant associations were also observed between NO_3_^−^ and NH_4_^+^ exposure and adverse health outcomes in preterm delivery in California [18]. PM_2.5_ components can also affect lung function [38]. It is probable that the causal relationship between DED and PM_2.5_ or its constituents is mediated by oxidative stress and chronic inflammation [39]. Studies have demonstrated that carbonaceous particles can induce pulmonary fibrosis through activation of the NLRP3 inflammasome [40–42]. Evidence suggested that neutrophil infiltration was related to the pathogenesis of NO_3_^–^ toxicity [43]. The possible mechanism by which sulfate mediates adverse health effects was thought to be related to the acidity of sulfate particles, which altered the toxicity of other constituents, rather than through its own direct toxicity [44].

Our findings that low temperatures increase the risk of outpatient visits for air pollution-related DED are biologically plausible. Low temperatures worsen contamination levels, and the cold environment affects the body’s immune function [21, 45]. The potential risk associated with SO_2_, NO_2_, and O_3_ on hospitalizations for cardiovascular diseases (CVDs) may be substantially heightened by low temperatures [22]. Moreover, interactions between low temperatures and high-polluted NO₂ and PM_2.5_ exposure have been associated with gingivitis incidence and morning hypertension [21, 46]. In addition, prolonged exposure to low ambient temperatures may precipitate dry eye symptoms and contribute to the development of ocular surface dysfunction through a shared pathophysiological pathway affecting the tear evaporation rate [47].

Middle-aged and young people are at a high risk of DED due to their frequent commutes across urban areas for work, leading to prolonged exposure to severe air pollution. Specifically, some mobile pollution sources, such as nitrogen dioxide derived from vehicle emissions, may increase the risk of dry eye [48]. Upon seasonal analysis, our findings indicated that the increased DED outpatient visits by air pollutants were more significant in winter. This was potentially attributable to air pollutant concentrations being relatively higher in winter than in other seasons. Moreover, the cold temperatures during winter align with the significant effect of air pollutants at low temperatures. Cold weather also constricts blood vessels on the ocular surface, affecting tear secretion and distribution [49]. Low temperatures may lead to meibomian gland dysfunction, affecting the quality of tears [50]. At last, the air is dry in winter, which leads to faster evaporation of tears, easily triggering dry eye symptoms [51, 52].

This study possessed several advantages: First, the association between air pollutants, including PM_2.5_ components, and DED was investigated and simultaneously the cumulative lagged effect was evaluated. The combination of time-stratified case-crossover design and DLNM provided robust adaptability to evaluate this association. Second, we further explored the modifying effect of temperature on the associations between air pollutants and PM_2.5_ components and daily DED cases. Finally, we also explored the vulnerable population characteristics and high-risk periods, which may facilitate DED patients’ self-management.

This study also had some notable limitations. First, the single location restricted the generalizability of the findings. Future research should further conduct multicenter studies, encompassing cities and regions with varying levels of pollution, climatic characteristics, and developmental stages. Second, mean exposure concentration calculated by aggregating data from fixed monitoring sites may introduce bias in the assessment of individual exposure levels. Future studies could utilize individual sampling devices to measure exposure levels to air pollution and temperature more accurately. Finally, although our study illustrated that air pollution was positively associated with DED outpatient visits, it could not prove causality. Future research should explore the underlying mechanisms and conduct studies to establish causal relationships.

## Conclusion

Air pollutants (PM_2.5_, PM_10_, NO_2_, SO_2_ and CO) and PM_2.5_ constituents (NO_3_^−^ and NH_4_^+^) were associated with DED, which was enhanced at low temperatures. Individuals aged 21–40 constitute a relevant vulnerable population, and winter is the high-risk period. Authorities should take action to control pollutant emissions and remind the public to minimize outdoor activities during periods of low temperatures and high pollution levels. Future research should further explore strategies to mitigate the adverse effects of air pollution on dry eye disease, such as the use of air purifiers and improvements in diet and lifestyle, to determine whether these measures could reduce related health risks.

## Electronic supplementary material

Below is the link to the electronic supplementary material.

Supplementary Material 1

## Data Availability

Data use requires contacting corresponding author, not yet publicly available.

## Abbreviations

DED: Dry eye disease
DLNM: Distributed lag nonlinear models
AF: Attributable fraction
TAP: Tracking Air Pollution
BC: Black carbon
OM: Organic matter
df: degrees of freedom
RR: Relative risk
IQR: Interquartile range
ROS: Reactive oxygen species
CVDs: Cardiovascular diseases
