# Supplementary material for: Associations between air pollution and daily outpatient visits for dry eye disease and the effect modification of temperature
Source: BMC Public Health. 2025 Mar 27;25:1163. doi: 10.1186/s12889-025-22187-4 (PMC11951751; doi:10.1186/s12889-025-22187-4)
Supplement: Supplementary file 1 — Supplementary Material 1 [file 12889_2025_22187_MOESM1_ESM.docx]

**Supplementary Information**

**Associations between air pollution and daily outpatient visits for dry eye disease and the effect modification of temperature**

Dandan Chu ^1,2†^, Juan Chen ^3†^, Chenlu Yang ^1^, Yan Li ^4^, Mingwei Wang ^3, 5*^, Junzhe Bao ^1*^

^1^College of Public Health, Zhengzhou University, Zhengzhou, 450001, China.

^2^Henan Eye Hospital, Henan Provincial People’s Hospital, People’s Hospital of Zhengzhou University, Zhengzhou, 450003, China.

^3^Affiliated Hospital of Hangzhou Normal University, Hangzhou, 310015, China.

^4^Department of Infection control, Henan Provincial People’s Hospital, People’s Hospital of Zhengzhou University, Zhengzhou, 450003, China.

^5^Hangzhou Institute of Cardiovascular Diseases, Hangzhou, 310015, China.

**^*^Corresponding author.**

E-mail: Mingwei Wang ([wmu990556@hznu.edu.cn](mailto:wmu990556@hznu.edu.cn)), Junzhe Bao ([baojz@zzu.edu.cn](mailto:baojz@zzu.edu.cn)), Tel: +86 180 2715 1989.

**Author.**

E-mail: Dandan Chu ([cddkn1114@163.com)](mailto:cddkn1114@163.com)%20D) , Juan Chen ([chenjuan564453@163.com](mailto:chenjuan564453@163.com)) , Chenlu Yang ([yangchenlu@gs.zzu.edu.cn](mailto:yangchenlu@gs.zzu.edu.cn)), Yan Li ([liyan02050110@163.com).](mailto:liyan02050110@163.com).%20)

^†^ These authors contributed equally to this work.

| **Table S1** The correlation between meteorological factors, air pollution, and daily cases of DED outpatient visits during the study period. | | | | | | | | | | |
| --- | --- | --- | --- | --- | --- | --- | --- | --- | --- | --- |
| Variables | Temperature | Relative  humidity | Air  pressure | PM_2.5_ | PM_10_ | NO_2_ | SO_2_ | CO | O_3_ | Daily  cases |
| Temperature | 1.00 |  |  |  |  |  |  |  |  |  |
| Relative humidity | 0.01 | 1.00 |  |  |  |  |  |  |  |  |
| PM_2.5_ | -0.39^*^ | -0.13^*^ | 0.36^*^ | 1.00 |  |  |  |  |  |  |
| PM_10_ | -0.34^*^ | -0.27^*^ | 0.35^*^ | 0.94^*^ | 1.00 |  |  |  |  |  |
| NO_2_ | -0.48^*^ | -0.08^*^ | 0.47^*^ | 0.69^*^ | 0.74^*^ | 1.00 |  |  |  |  |
| SO_2_ | -0.36^*^ | -0.34^*^ | 0.39^*^ | 0.64^*^ | 0.64^*^ | 0.57^*^ | 1.00 |  |  |  |
| CO | -0.42^*^ | 0.12^*^ | 0.34^*^ | 0.77^*^ | 0.69^*^ | 0.66^*^ | 0.42^*^ | 1.00 |  |  |
| O_3_ | 0.52^*^ | -0.38^*^ | -0.41^*^ | -0.20^*^ | -0.12^*^ | -0.38^*^ | -0.22^*^ | -0.4^*^ | 1.00 |  |
| Daily cases | 0.12^*^ | -0.11^*^ | -0.08^*^ | -0.17^*^ | -0.09^*^ | -0.03^*^ | -0.29^*^ | -0.09^*^ | 0.26^*^ | 1.00 |
| ^*^p<0.01 Spearman correlation analysis was adopted. | | | | | | | | | | |

| **Table S2** RR (95% CI) of air pollution on DED outpatient visits at different lag days in different temperature strata. | | | | | | |
| --- | --- | --- | --- | --- | --- | --- |
| Lag days | PM_2.5_ | PM_10_ | NO_2_ | SO_2_ | CO | O_3_ |
| **0-2** |  |  |  |  |  |  |
| all | **1.04 (1.00, 1.08)** | **1.05 (1.01, 1.10)** | **1.10 (1.06, 1.15)** | **1.06 (1.01, 1.11)** | 1.03 (0.99, 1.07) | 0.98 (0.94, 1.03) |
| low temperature | **1.09 (1.04, 1.13)** | **1.10 (1.05, 1.15)** | **1.17 (1.11, 1.23)** | **1.07 (1.02, 1.13)** | **1.07 (1.02, 1.13)** | 0.98 (0.91, 1.06) |
| high temperature | 0.94 (0.88, 1.01) | 0.95 (0.88, 1.02) | 0.97 (0.90, 1.05) | 1.06 (0.99, 1.14) | 0.97 (0.91, 1.04) | 0.98 (0.94, 1.03) |
| **0-3** |  |  |  |  |  |  |
| all | **1.05 (1.00, 1.09)** | **1.06 (1.01, 1.11)** | **1.12 (1.07, 1.18)** | **1.08 (1.03, 1.14)** | **1.04 (1.00, 1.09)** | 0.98 (0.93, 1.02) |
| low temperature | **1.09 (1.04, 1.15)** | **1.11 (1.05, 1.16)** | **1.21 (1.14, 1.29)** | **1.09 (1.04, 1.16)** | **1.08 (1.03, 1.14)** | 0.97 (0.89, 1.04) |
| high temperature | 0.95 (0.88, 1.02) | 0.95 (0.88, 1.04) | 0.96 (0.89, 1.05) | 1.07 (0.99, 1.15) | 0.99 (0.92, 1.06) | 0.98 (0.93, 1.03) |
| **0-5** |  |  |  |  |  |  |
| all | 1.04 (0.99, 1.10) | 1.03 (0.96, 1.09) | **1.12 (1.06, 1.19)** | **1.10 (1.04, 1.16)** | 1.04 (0.99, 1.09) | 0.98 (0.93, 1.03) |
| low temperature | **1.08 (1.02, 1.14)** | **1.07 (1.01, 1.14)** | **1.21 (1.14, 1.30)** | **1.10 (1.03, 1.17)** | **1.07 (1.01, 1.14)** | 0.96 (0.88, 1.04) |
| high temperature | 0.97 (0.89, 1.06) | 0.98 (0.89, 1.08) | 0.96 (0.88, 1.06) | **1.09 (1.01, 1.18)** | 0.99 (0.92, 1.07) | 0.99 (0.94, 1.05) |
| PM_2.5_, fine particulate matter; PM_10_, inhalable particulate matter; O_3_, ozone; NO_2_, nitrogen dioxide; SO_2_, sulfur dioxide; CO, carbon monoxide | | | | | | |

| **Table S3** AF (95% CI) of air pollution on DED outpatient visits at different lag days in different temperature strata. | | | | | | |
| --- | --- | --- | --- | --- | --- | --- |
| Lag days | PM_2.5_ | PM_10_ | NO_2_ | SO_2_ | CO | O_3_ |
| **0-2** |  |  |  |  |  |  |
| all | **5.49 (0.54, 10.57)** | **7.15 (1.80, 12.35)** | **16.6 (10.25, 22.98)** | **8.58 (2.10, 14.51)** | 8.91 (‒2.12, 19.49) | ‒2.28 (‒8.96, 4.29) |
| low temperature | **12.83 (5.91, 18.76)** | **14.54 (7.46, 20.72)** | **30.15 (21.33, 37.41)** | **11.11 (2.95, 18.84)** | **20.39 (5.57, 31.60)** | ‒1.57 (‒10.50, 5.50) |
| high temperature | ‒7.22 (‒15.92, 1.57) | ‒6.46 (‒16.58, 2.32) | ‒4.11 (‒16.50, 7.40) | 6.90 (‒1.67, 13.71) | ‒7.79 (‒27.57, 7.96) | ‒3.05 (‒12.92, 5.56) |
| **0-3** |  |  |  |  |  |  |
| all | **6.42 (1.09, 11.56)** | **8.00 (2.60, 13.60)** | **18.65 (11.52, 25.21)** | **10.82 (3.92, 17.24)** | **12.28 (0.23, 22.86)** | ‒3.63 (‒11.40, 3.60) |
| low temperature | **13.91 (6.30, 20.50)** | **15.52 (8.11, 22.65)** | **34.06 (25.52, 42.15)** | **14.44 (5.43, 21.63)** | **22.67 (6.95, 34.92)** | ‒3.62 (‒12.45, 4.15) |
| high temperature | ‒6.21 (‒15.32, 2.99) | ‒5.63 (‒15.71, 4.05) | ‒5.40 (‒19.08, 6.58) | 7.68 (‒0.97, 15.03) | ‒3.10(‒24.60,14.66) | ‒3.44 (‒13.45, 5.81) |
| **0-5** |  |  |  |  |  |  |
| all | 5.76 (‒0.29, 12.09) | 6.59 (‒0.73, 13.17) | **18.80 (10.00, 25.79)** | **12.54 (4.86, 18.82)** | 10.72 (‒4.16, 23.07) | ‒3.22 (‒11.26, 4.12) |
| low temperature | **11.77 (2.46, 19.83)** | **11.48 (1.46, 19.43)** | **34.21 (24.00, 41.94)** | **15.10 (5.48, 23.87)** | **20.86 (3.02, 35.56)** | ‒4.30 (‒14.35, 4.45) |
| high temperature | ‒3.88 (‒14.77, 5.60) | ‒2.12 (‒13.52, 8.68) | ‒5.65 (‒20.59, 6.82) | **10.43 (1.38, 18.70)** | ‒1.86 (‒26.17,17.78) | ‒2.00 (‒13.01, 7.78) |

| **Table S4**  RR (95% CI) of air pollution on DED outpatient visits by changing the df of temperature and relative humidity from 2 to 4. | |
| --- | --- |
|  | RR (95% CI) |
| df=2 |  |
| PM_2.5_ | **1.05 (1.01, 1.10)** |
| PM_10_ | **1.06 (1.02, 1.11)** |
| NO_2_ | **1.12 (1.06, 1.18)** |
| SO_2_ | **1.08 (1.03, 1.14)** |
| CO | **1.05 (1.00, 1.09)** |
| O_3_ | 0.97 (0.93, 1.02) |
| df=3 |  |
| PM_2.5_ | **1.05 (1.00, 1.09)** |
| PM_10_ | **1.06 (1.01, 1.11)** |
| NO_2_ | **1.12 (1.07, 1.18)** |
| SO_2_ | **1.08 (1.03, 1.14)** |
| CO | **1.04 (1.00, 1.09)** |
| O_3_ | 0.98 (0.93, 1.02) |
| df=4 |  |
| PM_2.5_ | **1.05 (1.00, 1.09)** |
| PM_10_ | **1.06 (1.01, 1.11)** |
| NO_2_ | **1.12 (1.07, 1.18)** |
| SO_2_ | **1.08 (1.03, 1.14)** |
| CO | **1.04 (1.00, 1.09)** |
| O_3_ | 0.98 (0.93, 1.02) |
